# Association between SSNHL and Thyroid Diseases

**DOI:** 10.3390/ijerph17228419

**Published:** 2020-11-13

**Authors:** So Young Kim, Young Shin Song, Jee Hye Wee, Chanyang Min, Dae Myoung Yoo, Hyo Geun Choi

**Affiliations:** 1 CHA Bundang Medical Center, Department of Otorhinolaryngology-Head & Neck Surgery, CHA University, Seongnam 13496, Korea; sossi81@hanmail.net; 2 CHA Bundang Medical Center, Department of Internal Medicine, CHA University, Seongnam 13496, Korea; yssongmd@gmail.com; 3 Department of Otorhinolaryngology-Head & Neck Surgery, Hallym University College of Medicine, Anyang 14068, Korea; weejh07@hanmail.net; 4 Hallym Data Science Laboratory, Hallym University College of Medicine, Anyang 14068, Korea; joicemin@naver.com (C.M.); ydm1285@naver.com (D.M.Y.); 5 Graduate School of Public Health, Seoul National University, Seoul 08826, Korea

**Keywords:** hearing loss, sudden, hyperthyroidism, cohort Studies, case-control studies

## Abstract

The association between thyroid disease and sudden sensorineural hearing loss (SSNHL) has not been evaluated. We investigated the association of goiter, hypothyroidism, thyroiditis, and hyperthyroidism with sudden sensorineural hearing loss (SSNHL). Data from the Korean National Health Insurance Service-Health Screening Cohort were used. The 8658 SSNHL patients were matched in a 1:4 ratio with 34,632 controls for age, sex, and region of residence. Histories of goiter, hypothyroidism, thyroiditis, hyperthyroidism, and Levothyroxine medication were explored as possible factors influencing SSNHL development. Associations were estimated using conditional logistic regression analyses, adjusted for Levothyroxine medication use. Subgroup analyses were conducted according to age, sex, income, and region of residence. SSNHL patients had a higher rate of goiter occurrence (4.4% vs. 3.7 %, *p* = 0.001) and hypothyroidism (4.0% vs. 3.2 %, *p* < 0.001) than controls. Goiter and hypothyroidism were positively associated with SSNHL (adjusted OR =1.14 (95% CI =1.01–1.28), *p* = 0.043 for goiter and 1.17 (95% CI =1.03–1.33), *p* = 0.016 for hypothyroidism). In subgroup analyses, hypothyroidism or goiter was more prevalent in SSNHL patients than in controls. Lower-income subgroups showed associations of hypothyroidism and goiter with SSNHL. SSNHL patients were more likely to have goiter and hypothyroidism than normal individuals.

## 1. Introduction

Thyroid hormone regulates cochlear development and function [1,2]. Thyroid hormone receptors regulate the functional development of cochlea through fast-activating potassium conductance [1]. In addition to inner or outer hair cells, the myelination of the cochleovestibular nerve is also regulated by thyroid hormone receptors [2]. Therefore, hypothyroidism during developmental periods has been reported to induce sensorineural hearing loss [3,4]. Conversely, hyperthyroidism has also been associated with hearing loss, suggesting that tight control of this pathway is essential for normal development [5,6]. A case-control study reported higher hearing thresholds in patients with Graves’ disease [5]. Sympathetic over-activation and autoimmunity have been suggested to contribute to hearing loss in hyperthyroidism [5,6]. Thus, we postulate that both hyper- and hypothyroidism may associate with hearing loss through several potential pathogenic mechanisms. 

Sudden sensorineural hearing loss (SSNHL) is defined as sudden onset hearing loss, and approximately 90% of cases are idiopathic [7]. The incidence of SSNHL is between 5 and 27 cases per 100,000 person-years [8]. Multiple causes, including autoimmunity [9], vascular compromise [10], viral infection [11], and metabolic disorders [12] have been suggested to be associated with SSNHL. As thyroid diseases encompass a wide spectrum of etiologies and are related to both autoimmunity [13] and vascular compromise [14], previous clinical studies have reported a positive association between thyroid diseases and hearing loss [12,15]. However, most of the previous studies were based on a small number of cases and focused on only one type of thyroid disease, such as Graves’ disease [5] or Hashimoto’s thyroiditis [16]. Only one cross-sectional cohort study demonstrated that both hyper—and hypothyroidism were positively associated with SSNHL. However, they separately analyzed for each type of thyroid disease and did not concurrently consider various other types of thyroid disease.

In this study, we hypothesized that any type of thyroid disease could relate to SSNHL. As each type of thyroid disease has a high degree of collinearity with other types, their co-occurrence must be adjusted before assessment for association with SSNHL. Besides, because differences in gender and socioeconomic inequalities [17] have been reported to influence thyroid disease, these factors were analyzed using subgroup analyses.

## 2. Materials and Methods

### 2.1. Study Population

The ethics committee of Hallym University (IRB 2019-10-023) permitted this study. Written informed consent was not claimed by the Institutional Review Board. All analyses followed the guidelines and regulations of the Hallym University ethics committee. A meticulous description of The Korea National Health Insurance Service-Health Screening Cohort data is reported elsewhere [18]. 

### 2.2. Definition of Sudden Sensorineural Hearing Loss (Dependent Variable)

Sudden sensorineural hearing loss (SSNHL) was included when the patients were diagnosed with International Classification of Diseases ICD-10 codes H912 (Sudden sensorineural hearing loss) by audiometric examination (claim code: E6931-E6937, F6341-F6348) and steroids prescription.

### 2.3. Levothyroxine Medications Use (Independent Variable)

Patients were classified as Levothyroxine medication users if they had been prescribed Levothyroxine medications ≥3 months.

### 2.4. Definition of Goiter (Independent Variable)

Patients were classified with goiter if they were diagnosed with ICD-10 codes E04 (other nontoxic goiter). Among these, we selected patients who were treated more than twice.

### 2.5. Definition of Hypothyroidism (Independent Variable)

Patients were defined as having hypothyroidism if they were diagnosed with ICD-10 codes E02 (subclinical iodine-deficiency hypothyroidism) and E03 (other hypothyroidism). Among these, we selected patients who were treated more than twice.

### 2.6. Definition of Thyroiditis (Independent Variable)

Patients were assigned to the thyroiditis category if they were diagnosed with ICD-10 codes E06 (thyroiditis). Among these, we selected patients who were treated more than twice.

### 2.7. Definition of Hyperthyroidism (Independent Variable)

Hyperthyroidism was defined as a diagnosis of ICD-10 codes E05 (hyperthyroidism). Among these, we selected patients who were treated more than twice.

### 2.8. Participant Selection

**Figure 1:**
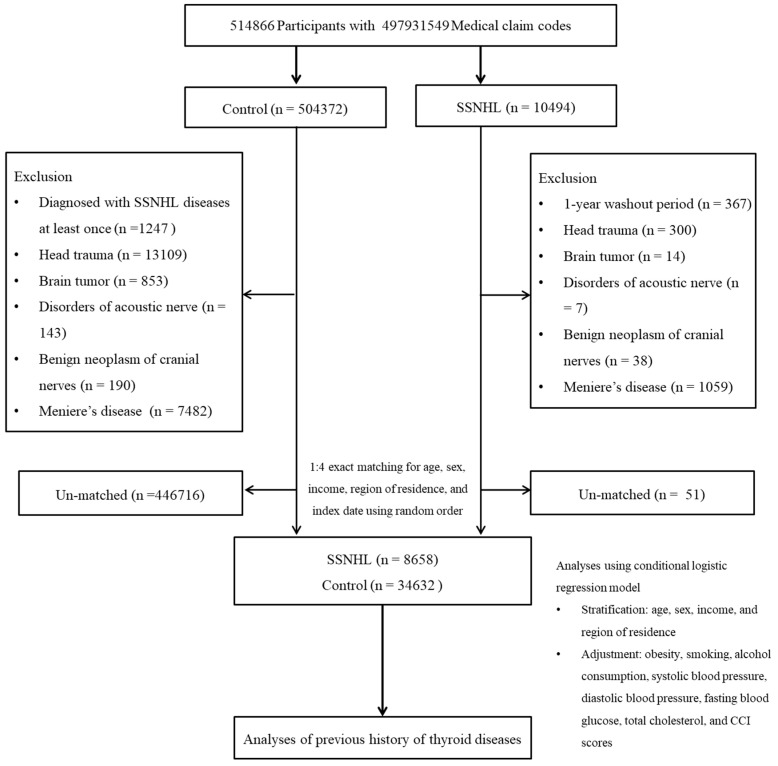
A schematic illustration of the participant selection process that was used in the present study. Of a total of 514,866 participants, 8658 of sudden sensorineural hearing loss (SSNHL) patients were 1:4 matched with 34,632 control participants for age, sex, income, and region of residence.

SSNHL patients were selected from 514,866 participants with 615,488,428 medical claim codes from 2002 through 2015 (*n* = 10,494). The control group included participants who were not diagnosed with SSNHL from 2002 through 2015 (*n* = 504,372). To select first time SSNHL patients only, those diagnosed in 2002 were excluded (washout periods, *n* = 367). The diagnosis of SSNHL in 2002 might contain the possibility of inclusion of participants who were diagnosed before 2002, in that they might have been followed up since 2000 or 2001. Therefore, we excluded participants from 2002. The SSNHL in 2003 would be the first diagnosed in that they have had one year of washout periods. In control participants, those who were diagnosed with SSNHL once were excluded (*n* = 1247). Participants with a history of head trauma or with head and neck Computed Tomography (CT) evaluations were excluded (ICD-10 codes: S00 to S09, diagnosed by neurologists, neurosurgeons, or emergency medicine doctors) (Claim codes: HA401-HA416, HA441-HA443, HA451-HA453, HA461-HA463, or HA471-HA473) (*n* = 300 for SSNHL, *n* = 13,109 for control). Participants who were treated for brain tumors ≥2 times (ICD-10 codes: C70 to C72) (*n* = 14 for SSNHL, *n* = 853 for control), disorders of the acoustic nerve ≥2 times (ICD-10 codes: H933) (*n* = 7 for SSNHL, *n* = 143 for control), and benign neoplasm of the cranial nerves ≥2 times (ICD-10 codes: D333) (*n* = 38 for SSNHL, *n* = 190 for control) were also excluded. Participants who were treated for Meniere’s disease ≥2 times (ICD-10 codes: H810) and underwent audiometric examinations were excluded (claim code: E6931-E6937, F6341-F6348) (*n* = 1059 for SSNHL, *n* = 7482 for control). SSNHL patients were 1:4 matched with control participants for age, gender, income, and region of residence. Because this study is based on medical claim data, the accessibility of medical facilities should be equalized to attenuate the possible bias due to the number of clinical visits. Thus, gender, income, and region of residence were matched between SSNHL and control participants. Age was matched to prevent the influence of age-related hearing loss. To minimize selection bias, the control participants were selected randomly. The index date of each SSNHL patient was set as the time of treatment of SSNHL. The index date of each SSNHL patient was set as the time of first treatment date and the diagnosis of SSNHL during 2003 through 2015.Therefore, each matched SSNHL patient has the same index date as their control. During the matching procedure, 51 SSNHL patients and 446,716 of control participants were excluded. Ultimately, 8658 SSNHL patients were matched in a ratio of 1:4 with 34,632 control participants (Figure 1).

### 2.9. Covariates

Age groups were divided into 5-year intervals: 40–44, 45–49, 50–54…, and 85+ years old (total of 10 age groups) [19]. Income groups were classified as 5 classes (class 1 (lowest income)—5 (highest income)) [19]. Region of residence was grouped into urban and rural areas following our previous study [19].

Tobacco smoking was categorized based on self-declared current smoking status (nonsmoker, past smoker, and current smoker) [19]. Self-declared alcohol consumption was categorized based on the frequency of alcohol consumption (<1 time a week and ≥1 time a week) [19]. Obesity was categorized by body mass index (BMI, kg/m^2^) as underweight, normal, overweight, obese I, and obese II following previous guidelines [20]. Missing BMI (23 / 43,290 (0.053%)) was replaced by mean values of variables from the final participants. The Charlson Comorbidity Index (CCI) was used to measure 17 comorbidities as the continuous variable (0 through 29 points) [21,22]. We excluded cancer and metastatic cancer from the CCI score.

Disorders of vestibular function (ICD-10 codes: H811, H812, and H813), and thyroid cancer (ICD-10 codes: C73) were additionally assigned if SSNHL patients had been treated more than twice.

### 2.10. Statistical Analyses

General characteristics between the SSNHL patients and the control group were compared using Chi-square tests [19].

To analyze odds ratios (ORs) with 95% confidence intervals (CIs), conditional logistic regression models for thyroid diseases in SSNHL were calculated. Crude, model 1 (adjusted for obesity, smoking, alcohol consumption, disorders of vestibular function, thyroid cancer, and CCI scores), and model 2 (stepwise selection method for model 1) conditional logistic regressions were generated. The analyses were stratified by age, gender, income, and region of residence. 

For the subgroup analyses, we divided participants by age and gender (<60 years old and ≥60 years old; men and women) and by income and region of residence (low and high; urban and rural) using crude, model 1 and model 2.

Two-tailed analyses were performed and significance defined as P values less than 0.05. SAS v 9.4 (SAS Institute Inc., Cary, NC, USA) was used for statistical analyses.

## 3. Results

SSNHL patients had a higher rate of goiter and hypothyroidism than the control group (4.4% vs. 3.7%, *p* = 0.001 for goiter and 4.0% vs. 3.2%, *p* < 0.001 for hypothyroidism, Table 1). The SSNHL group had a higher prevalence of Levothyroxine use compared to the control group (3.5% vs 2.9%, *p* = 0.002). The rate of thyroiditis and hyperthyroidism were not different between the SSNHL and the control group. Obesity, smoking status, and alcohol consumption were different between the SSNHL and the control group (all *p* < 0.001).

SSNHL patients had an increased risk of Levothyroxine medication use, goiter, and hypothyroidism in model 1 (adjusted OR = 1.26, 95% CI = 1.08–1.46, *p* = 0.003 for Levothyroxine medication use; adjusted OR = 1.19, 95% CI = 1.05–1.34, *p* = 0.007 for goiter; adjusted OR = 1.22, 95% CI = 1.08–1.39, *p* = 0.002 for hypothyroidism, Table 2, Table S1). In the final model, SSNHL patients had an increased risk of goiter and hypothyroidism (adjusted OR = 1.14, 95 % CI = 1.01–1.28, *p* = 0.043 for goiter and adjusted OR = 1.17, 95% CI = 1.03–1.33, *p* = 0.016 for hypothyroidism).

**Figure 2:**
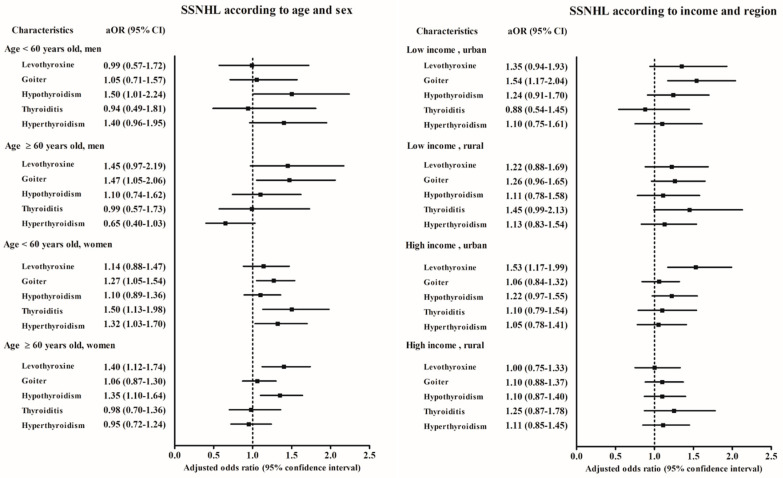
Odds ratios (95% confidence interval) of levothyroxine medication, goiter, hypothyroidism, thyroiditis, and hyperthyroidism for sudden sensorineural hearing loss according to age and sex and income and region of residence.

When considering age and gender, SSNHL patients with hypothyroidism were more common in patients who were <60 years old men, and ≥60 years old women (adjusted OR = 1.51, 95% CI = 1.01 –2.25, *p* = 0.044 for the <60 years old men group, and adjusted OR = 1.33, 95% CI = 1.10–1.62, *p* = 0.004 for the ≥60 years old women group, Table S2 and Figure 2). 

SSNHL patients were also more likely to have goiter in the ≥60 years old men and <60 years old women subgroups in the final model (adjusted OR = 1.45, 95% CI = 1.02–2.06, *p* = 0.037 for the ≥60 years old men group, and adjusted OR = 1.21, 95% CI = 1.00–1.46, *p* = 0.048 for the <60 years old women group, Table S2 and Figure 2).

SSNHL patients were also more likely to have goiter when subdividing for income and region of residence (adjusted OR = 1.50, 95% CI = 1.14–1.97, *p* = 0.004 low income and urban residence group. Table S3 and Figure 2). Hypothyroidism in SSNHL was increased in the low income, rural subgroup in the final model (adjusted OR = 1.37, 95% CI = 1.05–1.78, *p* = 0.019).

## 4. Discussion

SSNHL patients in the Korean National Health Insurance Service-Health Screening Cohort were more likely to have a history of either goiter or hypothyroidism than controls. These associations remained after adjusting for other types of thyroid diseases and Levothyroxine medication use and was present in both younger and older adults of both genders. SSNHL patients with goiter and hypothyroidism were more likely to belong to the low-income subgroup. The present study has extended previous findings by adjusting for lifestyle factors (obesity, smoking, alcohol consumption), and various types of thyroid diseases, and Levothyroxine medication use. To our knowledge, it is the biggest cohort study investigating the association between thyroid diseases and SSNHL. Although there is a lack of pathophysiological study on the association of hypothyroidism with SSNHL, a few previous studies have suggested a positive association between thyroid disorders and SSNHL [12,15,23]. A cross-sectional study by Oiticica et al. described a high prevalence of abnormal thyroid function in SSNHL patients (21.6%, 32/166) compared to the general population (21.6% (32/166) vs. 10%, *p* = 0.013) [12]. However, they did not discriminate between hyper or hypothyroidism. Both hyperthyroidism and hypothyroidism have been suggested to be associated with SSNHL [15,23]. Another cross-sectional study reported that 15.4% of SSNHL patients had low levels of Thyroid Stimulating Hormone (TSH) which was associated with poor prognosis [23]. A national health claim cohort study in Taiwan reported that hypothyroidism and hyperthyroidism were associated with a 1.54 fold (95% CI = 1.02–2.32, *p* = 0.042) and 1.41 fold (95% CI = 1.07–1.85, *p* = 0.045) higher odds for SSNHL, respectively [15]. However, they did not adjust for thyroid diseases and lifestyle factors such as obesity, smoking, and alcohol consumption. Plausible pathophysiological explanations for the association of thyroid diseases and SSNHL may include direct effects of thyroid hormone on the inner ear and indirect effects of thyroid hormone through the vasculature or on metabolic disturbances. 

A direct signaling pathway from thyroid hormone to inner ear spiral ganglion cells and inner and outer hair cells has been suggested. Triiodothyronine α receptor expression has been observed in spiral ganglion cells, and inner and outer hair cells in the rat cochlea [24]. In addition, in mouse models, hypothyroidism impaired the pruning of both type I and type II spiral ganglion neurons and delayed the attachment of efferent neurons to outer hair cells in the cochleae [25,26]. Thyroid hormone also regulated the integrity of the cochlear cytoskeleton [27]. Hypothyroidism disrupted the outer hair cell morphology and reduced microtubules in cochlear supporting pillar cells via decreased expression of fibroblast growth factor receptors and phosphorylated Cofilin [27]. The disruptions of these multiple signaling pathways between thyroid hormone and the inner ear may provide a link between thyroid dysfunction and SSNHL.

Thyroid diseases cause several systemic effects which may contribute to SSNHL including; autoimmune disease, vascular insufficiency, and electrolyte imbalance. Vascular compromise may lead to hearing loss, as generating endo-cochlear potential from ion transport via the stria vascularis is energetically expensive and, as the labyrinthine artery is an end artery, it is susceptible to any decreases in vascular function [28,29]. Deficiencies in antithrombin increases in homocysteine and factor VIII, and cardiovascular risk factors (arterial hypertension, hyperlipidemia, diabetes, and smoking) have previously been reported to be associated with SSNHL [30]. Hypercoagulability and increased risk of thromboembolism have been reported in hypothyroidism [31]. Thus, SSNHL patients may be at an increased risk of developing vascular insufficiencies driven by their thyroid diseases. Also, SSNHL patients may have an increased risk of autoimmune diseases such as systemic lupus erythematosus and rheumatoid arthritis [32,33]. Moreover, SSNHL patients may have an increased risk of electrolyte imbalances resulting from their thyroid diseases which may contribute to their hearing loss. The electrolyte imbalances of hypokalemia, hyperkalemia, and hypercalcemia have been reported in hyperthyroidism [34]. About 17%–50% of hyperthyroid patients were estimated to be hypercalcemic [35]. Because the maintenance of electrolyte composition of endolymph and perilymph is crucial to the correct conduction of sound in the inner ear, the disturbance of these electrolytes could induce inner ear dysfunction. Abnormal serum potassium levels were also reported to be concomitant with hearing loss in several cases [36,37]. In one instance, a patient with thyrotoxicosis developed SSNHL, thought to be due to hypokalemia and vascular compromise [37]. 

The present study used a large, representative population cohort which provided a sufficient number of control participants who were matched for age, gender, income, and region of residence. Lifestyle factors including obesity, smoking, and alcohol consumption as well as comorbidities were adjusted to minimize potential confounding effects. Besides, other thyroid diseases and Levothyroxine medications were considered because of the overlapping spectrum of thyroid diseases [38,39]. For example, a considerable number of hyperthyroidism patients experience hypothyroidism due to anti-thyroid treatments [38]. However, this study lacked results from thyroid function tests. Because the incidence of hypothyroidism fluctuates between 0.2% and 5.3% [40], the rate of hypothyroidism in SSNHL in this study is not significantly abnormal. In addition, the proportion of hypothyroidism was low in the SSHL patients (less than 5%). Therefore, this results should be interpreted with caution. For SSNHL, we meticulously excluded non-idiopathic causes of SSNHL including head trauma, brain tumor, disorders of the acoustic nerve, neoplasms of cranial nerves, and Meniere’s disease. In addition, the strict inclusion criteria relied on diagnosis by health system codes, pure tone audiometry tests, and steroid treatment. However, the degree and prognosis of hearing loss were not available in this study. Although many possible confounders were matched or adjusted in this study, there could be residual confounders, such as nutritional status, physical activities, sleep time, and stress. We could not attribute causality due to the cross-sectional study design. Future prospective studies using homogenous disease populations and pathophysiological studies could overcome the current limitations.

## 5. Conclusions

SSNHL was associated with goiter and hypothyroidism independent of other types of thyroid diseases and Levothyroxine medications. Both younger and older adult subgroups of SSNHL patients demonstrated a positive association with hypothyroidism.

## Figures and Tables

**Table 1:** General Characteristics of Participants.

Characteristics	SSNHL (*n*, %)	Control (*n*, %)
Age (years old)		
40–44	164 (1.9)	656 (1.9)
45–49	811 (9.4)	3244 (9.4)
50–54	1695 (19.6)	6780 (19.6)
55–59	1767 (20.4)	7068 (20.4)
60–64	1491 (17.2)	5964 (17.2)
65–69	1197 (13.8)	4788 (13.8)
70–74	872 (10.1)	3488 (10.1)
75–79	467 (5.4)	1868 (5.4)
80–84	166 (1.9)	664 (1.9)
85+	28 (0.3)	112 (0.3)
Sex		
Male	4560 (52.7)	18,240 (52.7)
Female	4098 (47.3)	16,392 (47.3)
Income		
1 (lowest)	1215 (14.0)	4860 (14.0)
2	1028 (11.9)	4112 (11.9)
3	1307 (15.1)	5228 (15.1)
4	1841 (21.3)	7364 (21.3)
5 (highest)	3267 (37.7)	13,068 (37.7)
Region of residence		
Urban	3818 (44.1)	15,272 (44.1)
Rural	4840 (55.9)	19,360 (55.9)
Obesity †*		
Underweight	157 (1.8)	818 (2.4)
Normal	2939 (34.0)	12,295 (35.5)
Overweight	2469 (28.5)	9509 (27.5)
Obese I	2871 (33.2)	10,933 (31.6)
Obese II	222 (2.6)	1077 (3.1)
Smoking status *		
Nonsmoker	6194 (71.5)	23,988 (69.3)
Past smoker	1253 (14.5)	4564 (13.2)
Current smoker	1211 (14.0)	6080 (17.6)
Alcohol consumption *		
<1 time a week	5702 (65.9)	22,405 (64.7)
≥1 time a week	2956 (34.1)	12,227 (35.3)
CCI score *		
0	6216 (71.8)	25,997 (75.1)
1	1543 (17.8)	5505 (15.9)
2	507 (5.9)	1726 (5.0)
3	221 (2.6)	778 (2.3)
≥4	171 (2.0)	626 (1.8)
Disorders of vestibular function *	1510 (17.4)	3163 (9.1)
Cerebrovascular diseases *	1463 (16.9)	5095 (14.7)
Thyroid cancer	77 (0.9)	299 (0.9)
Period of taking levothyroxine *		
<3month	8356 (96.5)	33,640 (97.1)
≥3month	302 (3.5)	992 (2.9)
Goiter *	380 (4.4)	1264 (3.7)
Hypothyroidism *	347 (4.0)	1119 (3.2)
Thyroiditis	145 (1.7)	485 (1.4)
Hyperthyroidism	220 (2.5)	796 (2.3)

Abbreviations: CCI, Charlson Comorbidity Index; SSNHL, Sudden sensorineural hearing loss. * Chi-square test. Significance at *p* < 0.05. ^†^ Obesity (BMI, body mass index, kg/m^2^) was categorized as < 18.5 (underweight), ≥18.5 to <23 (normal), ≥23 to <25 (overweight), ≥25 to <30 (obese I), and ≥30 (obese II).

**Table 2:** Crude and adjusted odd ratios (95% confidence interval) for SSNHL in levothyroxine, goiter, hypothyroidism, thyroiditis, and hyperthyroidism.

Characteristics	Odd Ratios for SSNHL					
	Crude ^†^	*p*-Value	Model 1 ^†,‡^	*p*-Value	Model 2 ^†,§^	*p*-Value
Total participants (*n* = 43,290)						
Levothyroxine	1.23 (1.08–1.40)	0.002 *	1.26 (1.08–1.46)	0.003 *		
Goiter	1.22 (1.08–1.37)	0.001 *	1.19 (1.05–1.34)	0.007 *	1.14 (1.01–1.28)	0.043 *
Hypothyroidism	1.26 (1.11–1.42)	<0.001 *	1.22 (1.08–1.39)	0.002 *	1.17 (1.03–1.33)	0.016 *
Thyroiditis	1.20 (1.00–1.45)	0.056	1.18 (0.97–1.42)	0.094		
Hyperthyroidism	1.11 (0.95–1.29)	0.182	1.10 (0.95–1.28)	0.218		

Abbreviations: CCI, Charlson Comorbidity Index; SSNHL, Sudden sensorineural hearing loss * Conditional logistic regression model, Significance at *p* < 0.05 ^†^ Models stratified by age, sex, income, and region of residence. ^‡^ Model 1 was adjusted for obesity, smoking, alcohol consumption, disorders of vestibular function, thyroid cancer, and CCI scores. ^§^ Model 2 was used stepwise selection method for model 1.

## Supplementary Materials

The following are available online at https://www.mdpi.com/1660-4601/17/22/8419/s1, Table S1: Pearson’s chi-square test between each of synthroid, goiter, hypothyroidism, thyroiditis, and hyperthyroidism, Table S2: Subgroup analyses of crude and adjusted odd ratios (95% confidence interval) for SSNHL in synthroid, goiter, hypothyroidism, thyroiditis, and hyperthyroidism according to age and sex, Table S3: Subgroup analyses of crude and adjusted odd ratios (95% confidence interval) for SSNHL in synthroid, goiter, hypothyroidism, thyroiditis, and hyperthyroidism according to income and region.
