# Supplementary material for: Association between SSNHL and Thyroid Diseases
Source: Int J Environ Res Public Health. 2020 Nov 13;17(22):8419. doi: 10.3390/ijerph17228419 (PMC7697232; doi:10.3390/ijerph17228419)
Supplement: Supplementary file 1 [file ijerph-17-08419-s001.pdf]

Table S1. Pearson's chi-square test between each of levothyroxine, goiter, hypothyroidism, thyroiditis, and hyperthyroidism.

		**Levothyroxine**	**Goiter**	**Hypothyroidism**	**Thyroiditis**	**Hyperthyroidism**
Levothyroxine	χ^2	1				
	p-value					
Goiter	χ^2	5869.4	1			
	p-value	<0.001 *				
Hypothyroidism	χ^2	18631.5	2398.9	1		
	p-value	<0.001 *	<0.001 *			
Thyroiditis	χ^2	2954.9	1320.7	1962.7	1	
	p-value	<0.001 *	<0.001 *	<0.001 *		
Hyperthyroidism	χ^2	2007.2	858.6	1472.0	691.8	1
	p-value	<0.001 *	<0.001 *	<0.001 *	<0.001 *	

* Chi-square test. Significance at $p < 0.05$.

Table S2. Subgroup analyses of crude and adjusted odd ratios (95% confidence interval) for SSNHL in levothyroxine, goiter, hypothyroidism, thyroiditis, and hyperthyroidism according to age and sex.

Characteristics	Odd Ratios for SSNHL					
	**Crude†**	***p*-Value**	**Model 1 †,‡**	***p*-Value**	**Model 2 †,§**	***p*-Value**
Age <60 years old, men (*n* = 11,905)						
Levothyroxine	0.78 (0.48–1.27)	0.318	0.99 (0.57–1.72)	0.973		
Goiter	1.02 (0.69–1.49)	0.937	1.05 (0.71–1.57)	0.788		
Hypothyroidism	1.50 (1.03–2.20)	0.036 *	1.50 (1.01–2.24)	0.043 *	1.51 (1.01–2.25)	0.044 *
Thyroiditis	1.07 (0.56–2.02)	0.842	0.94 (0.49–1.81)	0.860		
Hyperthyroidism	1.39 (0.99–1.97)	0.060	1.40 (0.96–1.95)	0.079		
Age ≥60 years old, men (*n* = 10,895)						
Levothyroxine	1.67 (1.16–2.41)	0.006 *	1.45 (0.97–2.19)	0.073	1.57 (1.05–2.34)	0.028 *
Goiter	1.63 (1.18–2.25)	0.003*	1.47 (1.05–2.06)	0.024 *	1.45 (1.02–2.06)	0.037 *
Hypothyroidism	1.21 (0.83–1.77)	0.312	1.10 (0.74–1.62)	0.640		
Thyroiditis	1.05 (0.60–1.82)	0.864	0.99 (0.57–1.73)	0.967		
Hyperthyroidism	0.69 (0.43–1.10)	0.120	0.65 (0.40–1.03)	0.068	0.57 (0.35–0.92)	0.021 *
Age <60 years old, women (*n* = 10,280)						
Levothyroxine	1.12 (0.90–1.40)	0.319	1.14 (0.88–1.47)	0.341		
Goiter	1.29 (1.07–1.55)	0.007 *	1.27 (1.05–1.54)	0.015 *	1.21 (1.00–1.46)	0.048 *
Hypothyroidism	1.11 (0.90–1.36)	0.326	1.10 (0.89–1.36)	0.379		
Thyroiditis	1.50 (1.14–1.98)	0.004 *	1.50 (1.13–1.98)	0.005 *	1.42 (1.07–1.88)	0.017 *
Hyperthyroidism	1.35 (1.05–1.73)	0.017 *	1.32 (1.03–1.70)	0.030*		
Age ≥60 years old, women (*n* = 10,210)						
Levothyroxine	1.34 (1.10–1.64)	0.004 *	1.40 (1.12–1.74)	0.004 *		
Goiter	1.09 (0.89–1.33)	0.408	1.06 (0.87–1.30)	0.575		
Hypothyroidism	1.37 (1.13–1.66)	0.002 *	1.35 (1.10–1.64)	0.004 *	1.33 (1.10–1.62)	0.004 *
Thyroiditis	0.98 (0.71–1.36)	0.921	0.98 (0.70–1.36)	0.889		
Hyperthyroidism	0.94 (0.72–1.24)	0.664	0.95 (0.72–1.24)	0.698		

Abbreviations: CCI, Charlson comorbidity index; SSNHL, Sudden sensorineural hearing loss. * Conditional logistic regression model, Significance at $p < 0.05$ † Models stratified by age, sex, income, and region of residence. ‡ Models adjusted for obesity, smoking, alcohol consumption, disorders of vestibular function, thyroid cancer, and CCI scores. § Model 2 was used stepwise selection method for model 1.

Table S3. Subgroup analyses of crude and adjusted odd ratios (95% confidence interval) for SSNHL in levothyroxine, goiter, hypothyroidism, thyroiditis, and hyperthyroidism according to income and region.

Characteristics	Odd Ratios for SSNHL					
	Crude†	*p*-Value	Adjusted †,‡	*p*-Value	Model 2 †,§	*p*-Value
	Low income, urban (*n* = 7340)					
Levothyroxine	1.25 (0.91–1.74)	0.171	1.35 (0.94–1.93)	0.104		
Goiter	1.59 (1.21–2.08)	<0.001 *	1.54 (1.17–2.04)	0.002 *	1.50 (1.14–1.97)	0.004 *
Hypothyroidism	1.28 (0.94–1.73)	0.115	1.24 (0.91–1.70)	0.180		
Thyroiditis	0.91 (0.56–1.48)	0.697	0.88 (0.54–1.45)	0.616		
Hyperthyroidism	1.11 (0.76–1.62)	0.605	1.10 (0.75–1.61)	0.634		
	Low income, rural (*n* = 10,410)					
Levothyroxine	1.22 (0.92–1.62)	0.177	1.22 (0.88–1.69)	0.228		
Goiter	1.29 (0.99–1.68)	0.057	1.26 (0.96–1.65)	0.095		
Hypothyroidism	1.42 (1.09–1.84)	0.009 *	1.38 (1.05–1.81)	0.022 *	1.37 (1.05–1.78)	0.019 *
Thyroiditis	1.51 (1.03–2.20)	0.004 *	1.45 (0.99–2.13)	0.057		
Hyperthyroidism	1.14 (0.84–1.54)	0.403	1.13 (0.83–1.54)	0.424		
	High income, urban (*n* = 11,750)					
Levothyroxine	1.42 (1.13–1.79)	0.003 *	1.53 (1.17–1.99)	0.002 *	1.37 (1.09–1.73)	0.008 *
Goiter	1.09 (0.87–1.35)	0.456	1.06 (0.84–1.32)	0.639		
Hypothyroidism	1.28 (1.02–1.60)	0.032 *	1.22 (0.97–1.55)	0.091		
Thyroiditis	1.11 (0.79–1.54)	0.555	1.10 (0.79–1.54)	0.565		
Hyperthyroidism	1.07 (0.80–1.43)	0.672	1.05 (0.78–1.41)	0.746		
	High income, rural (*n* = 13,790)					
Levothyroxine	1.05 (0.00–1.34)	0.702	1.00 (0.75–1.33)	0.995		
Goiter	1.13 (0.91–1.40)	0.277	1.10 (0.88–1.37)	0.424		
Hypothyroidism	1.12 (0.90–1.41)	0.317	1.10 (0.87–1.40)	0.417		
Thyroiditis	1.29 (0.90–1.84)	0.164	1.25 (0.87–1.78)	0.229		
Hyperthyroidism	1.13 (0.86–1.47)	0.383	1.11 (0.85–1.45)	0.443		

Abbreviations: CCI, Charlson comorbidity index; SSNHL, Sudden sensorineural hearing loss. * Conditional logistic regression model, Significance at $p < 0.05$. † Models stratified by age, sex, income, and region of residence. ‡ Models adjusted for obesity, smoking, alcohol consumption, disorders of vestibular function, thyroid cancer, and CCI scores. § Model 2 was used stepwise selection method for model 1.
